# Superconducting critical temperature elevated by intense magnetic fields

**DOI:** 10.1073/pnas.2422156122

**Published:** 2025-01-08

**Authors:** Z. Wu, H. Chen, T. I. Weinberger, A. Cabala, D. E. Graf, Y. Skourski, W. Xie, Y. Ling, Z. Zhu, V. Sechovský, M. Vališka, F. M. Grosche, A. G. Eaton

**Affiliations:** ^a^Cavendish Laboratory, Department of Physics, University of Cambridge, Cambridge CB3 0HE, United Kingdom; ^b^Faculty of Mathematics and Physics, Department of Condensed Matter Physics, Charles University, Prague 2 121 16, Czech Republic; ^c^National High Magnetic Field Laboratory, Tallahassee, FL 32310; ^d^Hochfeld-Magnetlabor Dresden (HLD-EMFL), Helmholtz-Zentrum Dresden-Rossendorf, Dresden 01328, Germany; ^e^Wuhan National High Magnetic Field Center, Wuhan 430074, China

**Keywords:** triplet superconductor, high magnetic field, heavy fermion

## Abstract

Below a critical temperature $T_{c}$, superconductors transport electrical charge without dissipative energy losses. The application of a magnetic field B generally acts to suppress $T_{c}$, up to some critical field strength at which $T_{c}\to$ 0 K. Here, we investigate magnetic field–induced superconductivity in high-quality specimens of the triplet superconductor candidate UTe_2_ in pulsed magnetic fields up to B = 70 T. Strikingly, we find that this material has a higher $T_{c}$ when B> 40 T ($T_{c}\approx$ 2.4 K) than it does for B= 0 T ($T_{c}=$ 2.1 K). This observation points to a fundamentally distinct mechanism for the formation of superconductivity at high B in UTe_2_ compared to the case of B = 0 T.

The heavy fermion paramagnet UTe_2_ exhibits numerous signatures of odd-parity (spin-triplet) superconductive pairing. These include high upper critical field strengths in excess of the Pauli pair-breaking limit for all orientations of B, along with only small changes in the NMR Knight shift upon crossing $T_{c}$ (1, 2). Initial investigations of the superconductive properties of UTe_2_ studied samples grown by the chemical vapor transport technique with typical values of $T_{c}\approx$ 1.6 K (3); subsequent optimization of a salt-flux growth technique has yielded higher quality specimens with $T_{c}$ = 2.1 K (4, 5). Remarkably, under the application of large magnetic fields, UTe_2_ exhibits two field-induced superconductive states (6). Thermodynamic evidence suggests that these phases are distinct from the ground state superconductivity found at B= 0 T (7, 8). The three superconductive phases of UTe_2_ are typically referred to as SC1, SC2, and SC3. The highest field superconducting state (SC3) has been found to persist up to B≈ 70 T (9) and is acutely sensitive to the orientation of B. SC3 has been found to demonstrate a remarkable resilience against the introduction of crystalline disorder (10). Recent measurements have pointed toward the presence of quantum critical fluctuations at very high B as a likely explanation for this exotic superconductivity (11), reminiscent of the case of the ferromagnetic superconductor URhGe (12).

## Results and Discussion

Here, we investigate the sensitivity to temperature and magnetic field tilt angle of the high-B SC3 superconducting phase in pristine quality UTe_2_. We performed contacted and contactless electrical conductivity measurements (*Materials and Methods*) in steady and pulsed magnetic fields, up to a maximal value of B= 70 T. Measurements were performed at magnetic field tilt angles $\theta_{b-c}$, defined as the angle of rotation from the crystallographic b axis toward the c axis.

**Fig. 1.**
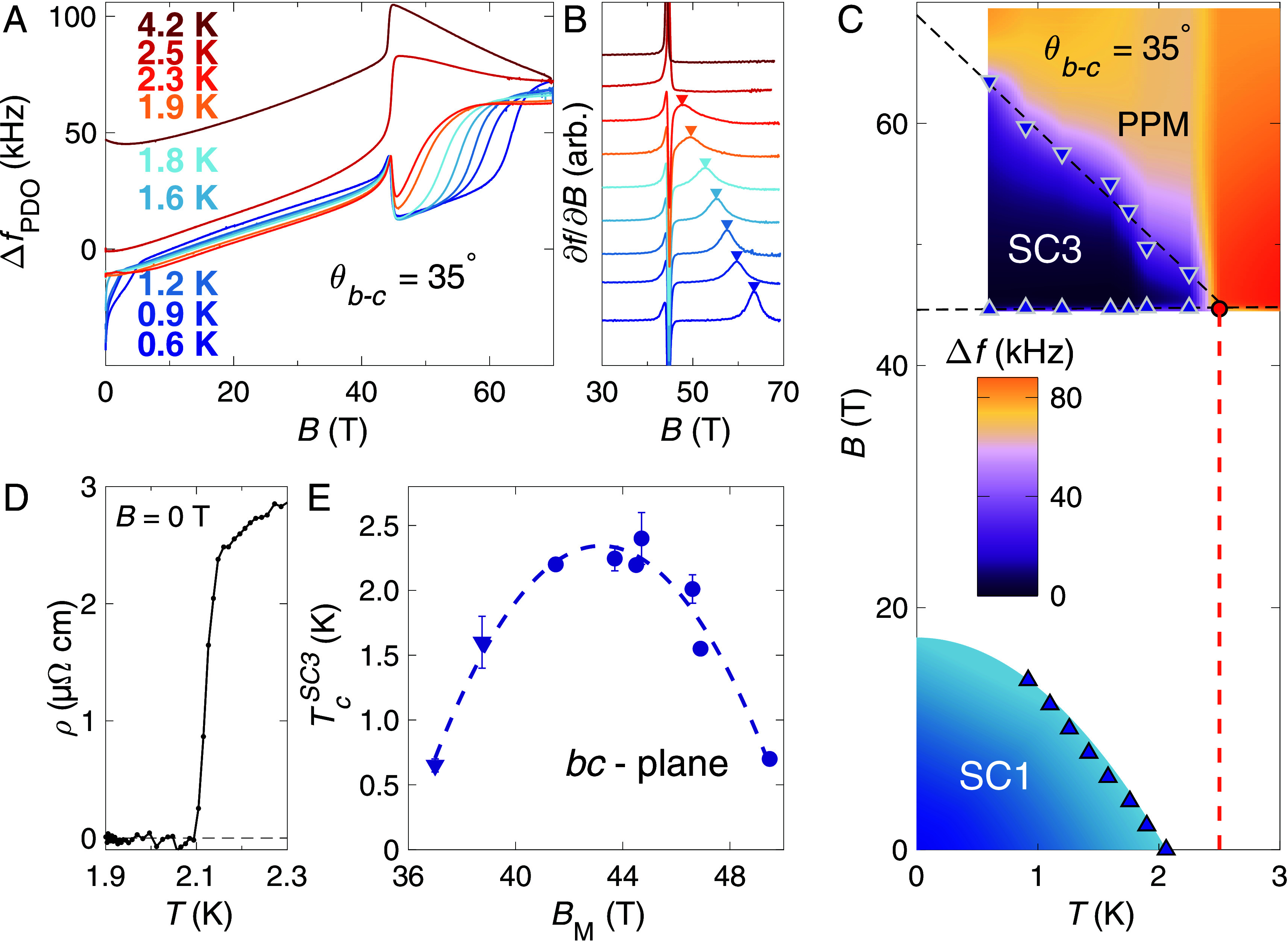
Enhancement of $T_{c}^{SC3}$ above $T_{c}^{SC1}$. (*A*) Pulsed field contactless conductivity measurements by the PDO technique (*Materials and Methods*) at incremental temperatures as indicated. The pronounced jump in the PDO signal at B≈ 45 T is due to a sudden increase in resistivity upon entering the polarized paramagnetic (PPM) state at the two highest measured temperatures, and due to the sudden onset of zero resistivity at all other (lower) temperatures as the SC3 state is accessed. (*B*) Derivatives with respect to B of the data from panel (*A*). (*C*) The phase diagram of SC1 and SC3 for $\theta_{b-c}=$ 35^°^, using the data from panel (*A*) to define the SC3 region. Note that the SC2 phase is not present at this tilt orientation of B(5). Dashed lines are given as a guide to the eye. Points for the termination of SC3 are taken from the arrows in panel (*B*) and plotted as “down” triangles, while “up” triangles are from the sharp onset of SC3 located at B≈ 45 T for each of these temperatures. The extrapolation indicates that $T_{c}^{SC3}\approx$ 2.4 K for this orientation of B. (*D*) Contacted resistivity measurement at B = 0 T showing that $T_{c}^{SC1}=$ 2.1 K. (*E*) The dome-like angular profile of $T_{c}^{SC3}$ plotted as a function of metamagnetic transition field $B_{\;\text{M}}$ for rotations in the b−c plane, in which $B_{\;\text{M}}$ evolves as $1/\cos\theta_{b-c}$ (9). Triangular points are from contacted transport in steady fields, with circular symbols from contactless conductivity measurements in pulsed fields.

We find that the maximal value of $T_{c}^{SC3}$ is reached for $\theta_{b-c}\approx$ 35^°^. In Fig. 1*A* we plot contactless conductivity measurements at this orientation of B for incremental temperatures. The transition to the SC3 phase is identified by a sharp dip in the derivative of the signal. This dip is still clearly visible at T= 2.3 K but is gone at T = 2.5 K. By plotting the extent of SC3 in B and T in Fig. 1*C*, the dashed line extrapolates to indicate $T_{c}^{SC3}\approx$ 2.4 K for this orientation of B. This is remarkable, given that for B = 0 T, $T_{c}^{SC1}$ = 2.1 K (Fig. 1*D*).

**Fig. 2.**
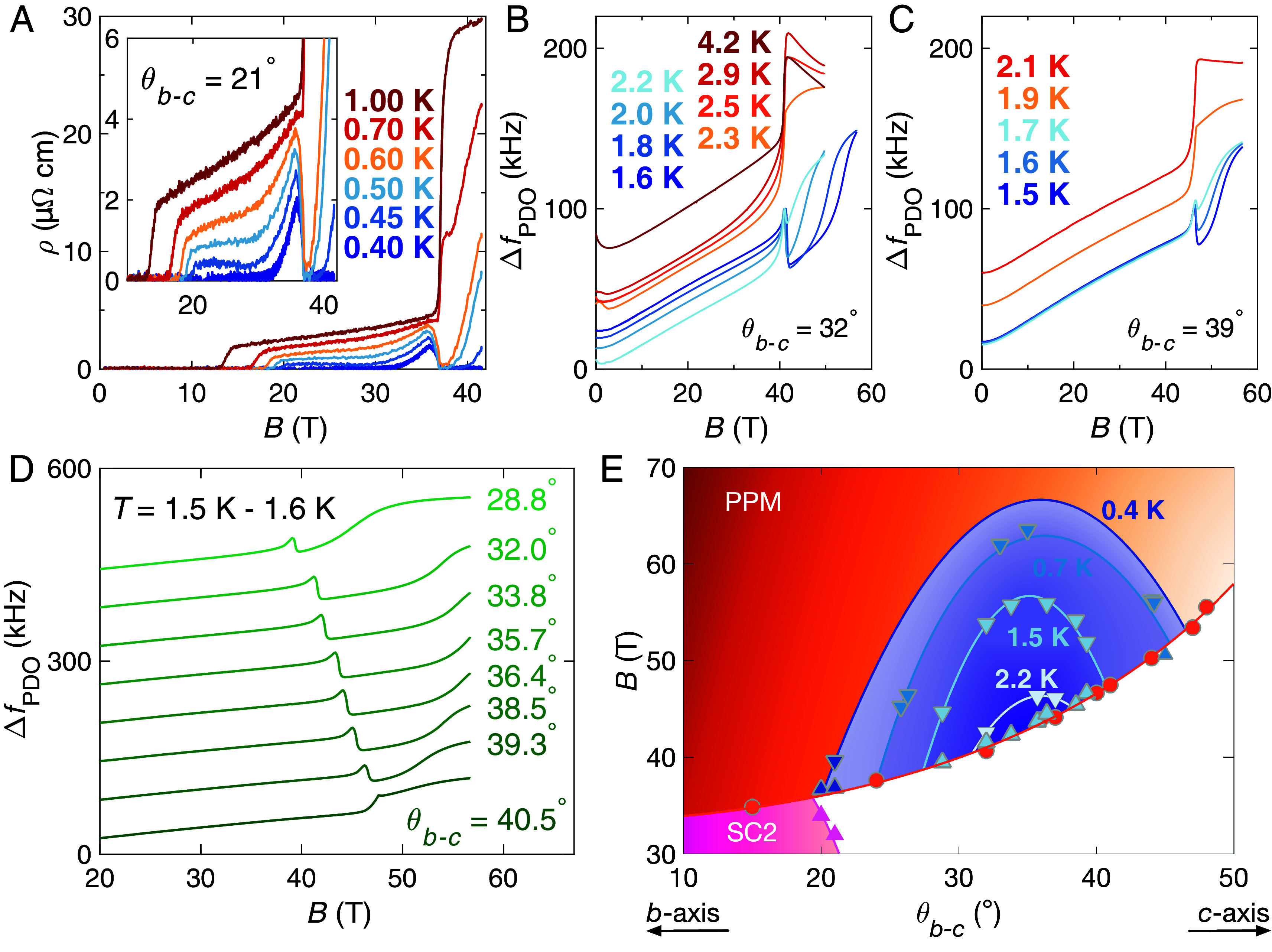
Angular evolution of $T_{c}^{SC3}$. (*A*) Contacted resistivity data measured in static fields showing that at $\theta_{b-c}=$ 21^°^, $T_{c}^{SC3}\approx$ 0.6 K. The sharp upturn in ρ is characteristic of accessing the PPM state (in the absence of coexisting superconductivity). (*B*) Pulsed field contactless conductivity data showing that at $\theta_{b-c}=$ 32^°^$T_{c}^{SC3}\approx$ 2.2 K while (*C*) at $\theta_{b-c}=$ 39^°^, $T_{c}^{SC3}\approx$ 1.8 K. (*D*) Isothermal rotation data showing the termination of SC3 for $\theta_{b-c}⪆$ 40^°^ at T≈ 1.5 K. (*E*) Angular phase diagram of UTe_2_ at high B, showing the temperature evolution of SC3 in the b−c rotational plane. The polarized paramagnetic (PPM) and SC2 phases are also indicated, with points from ref. 5. Isotherm curves indicating the temperature domain of SC3 are drawn as guides to the eye, with quoted temperatures accurate to within approximately 0.1 K. The 0.4 K isotherm is constrained by measurements in steady fields at low $\theta_{b-c}$, and extrapolated from the angular evolution of $T_{c}^{SC3}$ observed at higher T in our pulsed field experiments at high $\theta_{b-c}$, which we include for illustrative purposes. The evolution of $T_{c}^{SC3}$ with $\theta_{b-c}$ appears to be reasonably symmetric either side of $\theta_{b-c}$ = 35^°^.

We mapped the extent of SC3 at multiple orientations of B from $\theta_{b-c}=$ 21^°^ up to $\theta_{b-c}=$ 46^°^ (Fig. 2). We find that $T_{c}^{SC3}$ exhibits a dome-like angular dependence, being only ≈0.6 K at $\theta_{b-c}=$ 21^°^, extending up to ≈2.4 K at $\theta_{b-c}=$ 35^°^, and then reducing down to ≈1.8 K at $\theta_{b-c}=$ 39^°^. For $\theta_{b-c}\geq$ 45^°^ we find that the SC3 transition is no longer observable at 0.7 K, setting the upper boundary of the 0.7 K isotherm in Fig. 2*E*. We note that all temperatures quoted for pulsed field measurements were determined immediately before the pulse. These temperature values are therefore lower bounds on the actual temperature of the sample during the measurement, which may undergo some heating from eddy currents and vortex motion caused by the rapid rate of change of B.

The observation of superconducting critical temperature being elevated by the application of a magnetic field is highly unusual. In the case of the related material URhGe, the observation of $T_{c}(B=0$ T$)<T_{c}(B>0$ T) was associated with the proximity to a B-induced quantum critical end point (12) located at B∼ 10 T. What is remarkable about the case of UTe_2_ is that the high-B superconductivity extends up to B≈ 70 T (9). Preliminary experiments indicate that a similar mechanism of quantum criticality driving B-induced superconductivity appears to also be at play in UTe_2_ (11).

## Materials and Methods

UTe_2_ single crystals were grown in a salt flux (4) using the methodology detailed in ref. 13. Samples were screened for quality by a combination of residual resistivity, magnetic susceptibility, and specific heat capacity measurements. High-quality specimens were then oriented by X-ray Laue diffractometry.

Contacted electrical conductivity measurements were performed by the four-probe technique, with ac current sourced along the a direction at low frequencies (<50 Hz). Contactless conductivity was measured by the proximity detector oscillator (PDO) method, by the same methodology as our previous measurements reported in ref. 14. This involves tracking the change in frequency of the PDO circuitry, $\Delta f_{\;\text{PDO}}$, which relates to changes in the skin depth, resistivity, and susceptibility of the sample (15). All PDO data presented in this study were acquired on down-sweeps of magnetic field pulses, which possess a much more gradual rate of change of magnetic field strength with respect to time than the up-sweeps. Data from Hochfeldlabor-Dresden (HLD) were acquired on the same sample utilizing an identical experimental setup (but different angular orientation with respect to B) as that reported in our prior quantum interference study (14), where the excellent Lifshitz–Kosevich fitting of oscillatory amplitudes with respect to temperature gives strong confidence that the sample temperature remained close to equilibrium during the down-sweep of the magnetic field pulse.

## Data Availability

The datasets supporting the findings of this study are available from the University of Cambridge Apollo Repository (16).
